# Ubiquinol-cytochrome c reductase core protein 1 may be involved in delayed cardioprotection from preconditioning induced by diazoxide

**DOI:** 10.1371/journal.pone.0181903

**Published:** 2017-07-27

**Authors:** Zonghong Long, Guangyou Duan, Hong Li, Tingting Yi, Xiaoxiao Wu, Feng Chen, Zhuoxi Wu, Yuqi Gao

**Affiliations:** 1 Department of Anesthesiology, Xinqiao Hospital, Third Military Medical University, Chongqing, China; 2 Key Laboratory of High Altitude Medicine, Third Military Medical University, Chongqing, China; Indiana University School of Medicine, UNITED STATES

## Abstract

This study aimed to use long-term diazoxide treatment to establish a loss-of-cardioprotection model and then perform proteomics analysis to explore which proteins of mitochondrial inner membrane (MIM) are potentially involved in delayed cardioprotection. Rats received 1 to 8 weeks of diazoxide treatments (20 mg•kg^–1^•d^–1^) to establish a loss-of-cardioprotection model in different groups. Detection of serum cTnI levels and cell apoptosis assays in heart tissue were performed. Then, rats MIM after 0, 4 and 6 weeks of diazoxide treatment was isolated and proteomics analysis was performed. An invitro model of H9C2 cells was performed to explore the effects of targeted protein on delayed cardioprotection. The effect of delayed cardioprotection by diazoxide preconditioning disappeared when diazoxide treatments were given for six weeks or longer. Ubiquinol-cytochrome c reductase core protein 1 (UQCRC1) was identified in the proteomics analysis. UQCRC1 expression was upregulated by diazoxide treatment in H9C2 cells, and UQCRC1 down-regulation could increase the lactate dehydrogenase release and apoptosis rate after injury induced by oxygen glucose deprivation. These results showed that UQCRC1 might contribute to the loss-of-cardioprotection model induced by long-term diazoxide treatment and play a role in delayed cardioprotection.

## Introduction

Ischemic preconditioning (IPC) can effectively protect cardiac cells against injury and reduce myocardial infarct size caused by a subsequent prolonged period of myocardial ischemia [1]. The protective effect lasts up to 3 hours (the first period or window of protection) and is also followed by a second window of protection (SWOP) that begins at approximately 24 hours and lasts until 72 hours after the initial preconditioning event [2]. This effect of ischemic preconditioning could be inhibited by K^+^ channel blockers and can also be mimicked by the K^+^ channel opener diazoxide, which potentially enhances K^+^ flux at the mitochondrial membrane [3, 4]. Thus, mitochondrial ATP sensitive potassium channels (mitoK_ATP_) have been linked to IPC and have been identified as critical mediators of cardioprotection [5, 6]. It can be speculated that mitochondrial proteins interacting with mitoK_ATP_ during IPC are likely to be involved in cardioprotection.

Although many previous studies have identified protein expression changes after IPC or induced IPC mimicked by adenosine, diazoxide and inhalation anesthetics, many of these studies have targeted mitochondria [7–11]. At present, proteins that directly relate to mitoK_ATP_ and play a role in cardioprotection have rarely been identified. As we know, mitoK_ATP_ is located in the mitochondrial inner membrane (MIM) and some proteins in the MIM have recently been identified as targets for decreasing cardiac ischemia/reperfusion injury [12]. Additionally, using MIM samples, a previous study used proteomic analysis of the MIM to successfully identify a pore-forming subunit of the mitoK_ATP_ channel [13]. Nevertheless, the constitution of the mitoK_ATP_ channel remains unclear. Thus, the current study aimed to use the MIM sample combined with a proteomic approach to directly identify the proteins that are possibly involved in delayed cardioprotection.

In previous studies, a model of cardioprotection induced by IPC or some pharmacological application was used to explore possible functional proteins [12, 14, 15]. Interestingly, in contrast, several studies have demonstrated that the long-term administration of adenosine agonists ornicorandil, a mitoK_ATP_ channel opener, can abolish the effect of cardioprotection by ischemic and pharmacological preconditioning [16, 17]. This indicated that a long-term opening state might alter the expression of the functional proteins that are involved in cardioprotection. It is generally accepted that down-regulated proteins of the signal transduction pathway that underlies preconditioning might be an important mechanism of deprivation of preconditioning cardioprotection. Therefore, this provided a potential strategy to accurately investigate the MIM proteins that might be involved downstream of the mitoK_ATP_ action loop and that might contribute to delayed cardioprotection of preconditioning. Based on this information, the current study aimed to use long-term diazoxide treatment to establish a loss-of-cardioprotection model, then use two-dimensional flourescence differential gel electrophoresis (2D-DIGE) and MS analysis to investigate the MIM proteins that are potentially involved in delayed cardioprotection. An invitro model of H9C2 cardiac myocytes was also used to validate the effect of targeted proteins in cardioprotection. Through these approaches, the current study aimed to identify the possible targeted molecular sites that are involved in delayed cardioprotection.

## Materials and methods

### Animals

The animal protocol was approved by the Institutional Animal Ethics Committee, Third Military Medical University, Chongqing, China. A total of 105 adult male rats aged 4 to 6 weeks, weighing approximately 180–200 g, were used for the current study. The animals were housed according to the standards of specific pathogen free animals. All animal experiments were performed in accordance with the National Institutes of Health Guide for the Care and Use of Laboratory Animals (NIH publications number 80–23) revised in 2011.

### Animal protocol of long-term dizoxide treatment

**Fig 1:**
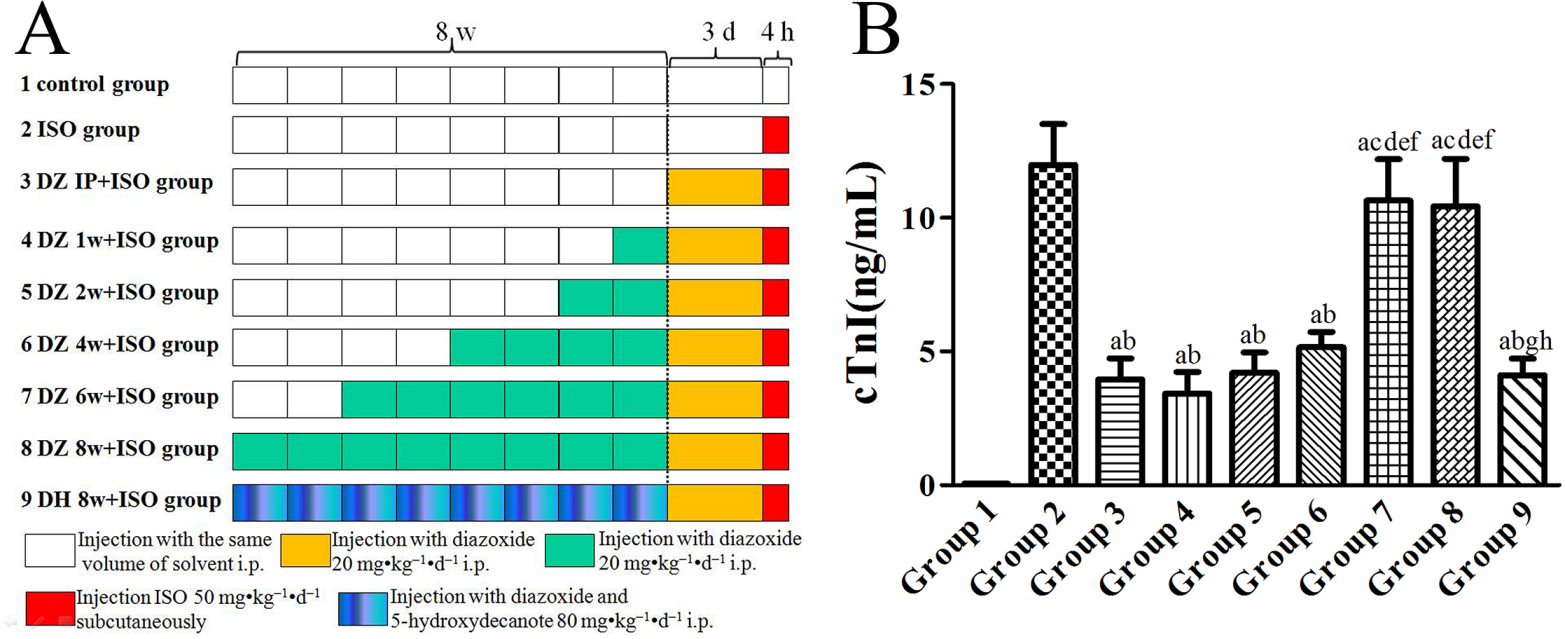
**The experimental schematic diagram (A) and changes in blood cTnI levels (B).** Data were expressed as mean ± SD, n = 10; a: *P* < 0.01 *vs*. group 1; b: *P* < 0.01 *vs*. group 2; c: *P* < 0.01 *vs*. group 3; d: *P* < 0.01 *vs*. group 4; e: *P* < 0.01 *vs*. group 5; f: *P* < 0.01 *vs*. group 6; g: *P* < 0.01 *vs*. group 7; h: *P* < 0.01 *vs*. group 8.

As shown in Fig 1A, ninety rats were randomly divided into 9 groups with 10 rats per group. Group 1 served as a control group. Group 2 served as an ISO (isoproterenol hydrochloride) group only treated with subcutaneous injection of ISO (50 mg•kg^−1^ dissolved in normal saline), and the rats were euthanized by cervical decapitation under halothane anesthesia at 4 h after ISO injection. The heart tissue and blood sample of these rats were collected. The rats in Group 3 were intraperitoneally preconditioned with dizoxide 20 mg•kg^–1^•d^–1^ for three days before ISO injection. Groups 4, 5, 6, 7 and 8 rats received 1, 2, 4, 6 and 8 weeks of pretreatment with diazoxide 20 mg•kg^–1^•d^–1^ (one injection per day), respectively. In all the other control periods, a comparable amount of normal saline was administered. In group 9, 5-hydroxydecanoic acid (5-HD, 80 mg•kg^–1^•d^–1^), a selective mitoK_ATP_ channel blocker [18, 19], was also given together with diazoxide for 8 weeks to explore whether the possible loss-of-cardioprotection effect could be avoided by inhibiting diazoxide.

### Detection of cardiac troponin I (cTnI) and apoptosis assessment

Triage-Examiner® (Biosite, USA) was applied to measure cTnI in rat serum samples of groups 1 to 9 using sandwich immune-fluorescence with double-antibodies. Then, based on the results of cTnI detection, we selected the segregate myocardial tissues of group 1, 2, 3, 6, 7, 8 and 9 for cell apoptosis assessment using TUNEL technology. A light microscope with normal spectra was used to perform microscopic observations. Five different fields from each sample were randomly observed, and the apoptosis rate was calculated according to the number of positive cells/the total number of cells × 100%.

### Animal protocol for proteomic analysis

Based on the above experiment of the current study, we performed proteomic analysis using the MIM to identify the proteins that possibly underlie the effects of 6 weeks of long-term diazoxide treatment, the time point at which the phenomenon of loss-of-cardioprotection appeared. Fifteen adult male rats were randomly divided into 3 groups, including the control group and two long-term diazoxide treatment groups, DZ 4w and DZ 6w. The rats in the DZ 4w and DZ 6w groups were intraperitoneally pretreated with dizoxide 20 mg•kg^–1^•d^–1^ for four and six weeks. The control group (DZ 0w) rats were injected with comparable amounts of normal saline.

### Isolation and purification of MIM

Mitochondrial samples were isolated and purified according to the previous reported method [20]. The cardiac ventricle tissues were separated, cubed, and minced in cooled sucrose buffer. The mince was diluted and homogenized and then mitochondria were isolated through gradient centrifugation. MIM was isolated and purified as described by Da Cruz S et al [21]. The purity of the mitochondria sample was assessed as described by Taylor SW et al [22]. First, antibodies against markers of the MIM (prohibitin), the endoplasmic reticulum (glucose regulated protein 94 kDa, GPR94), and peroxisomes (catalase) were used to compare the samples of crude mitochondria, purified mitochondria and MIM. Secondly, labeled anti- mHsp70 (located in mitochondrial matrix), anti-Bcl-X_L_ (located on outer mitochondrial membrane) and anti-COX IV (located on MIM) were used for assessing the purity of purified mitochondria, mitoplasts and MIM. Western blotting analysis was performed to quantify the expression of the different labeled proteins.

### 2D-DIGE technology

2D-DIGE technology as described by previous studies [23] was performed to detect the changes in protein abundance between different MIM samples of the DZ 0w group and the DZ 4w group and between the DZ 0w group and the DZ 6w group. Equal amounts of MIM proteins (5 μg/μL) from the DZ 0w group and the DZ 4w or DZ 6w groups were mixed to a final concentration of 50 μg/10 μL. Following the labeling reaction, 50 μg of each Cy2, Cy3 and Cy5 labeled samples were mixed. Then, after the labeled MIM samples were actively rehydrated and focused proteins were further separated on the 12.5% homogeneous SDS-PAGE gels. The SDS-PAGE gels were run in the second dimension at 15 mA/gel for 20 min and then at 30 mA/gel at 15°C until the bromophenol blue dye front reach the bottom of the gel.

A typhoon 9400 imager (Amersham Biosciences, Sweden) was used to visualize the labeled proteins. The digitized images were analyzed with the image master software DeCyder V4.0 (Amersham Biosciences, Sweden) using a 2-D analysis software platform designed specifically for use with DIGE. Spots with significant change across the groups were selected for subsequent identification.

### Protein identification and targeted protein validation

Significant protein spots were cut from the gels for comparing the DZ 6w group with the DZ 0w group. The gels were destained for 20 min in 30 mM potassium ferricyanide/100 mM sodium thiosulfate (1:1 v/v) and washed in Milli-Q water until the gels were destained. The spots were kept in 0.2 M NH_4_HCO_3_ for 20 min and then lyophilized. Each spot was digested overnight in 12.5 ng/mL trypsin in 0.1 M NH_4_HCO_3_. The peptides were extracted three times with 50% ACN, 0.1% TFA. The separation and identification of the digested protein were measured on a Micromass Tof Spec MALDI-TOF mass spectrometer (Manchester, UK). The PMF data from MALDI-TOF/MS were analyzed by searching NCBInr databases using MASCOT search software. In addition, western blotting analysis was performed to quantify the expression of UQCRC1 using the MIM sample from the rats of the group DZ 0w, group DZ 4w and group DZ 6w.

### Detection of UQCRC1 expression in H9C2 cells after diazoxide or 5-HD treatment

H9C2 cells were given different treatments: control group was treated with solvent, DZ-1h group with diazoxide 100 μmol/L for 1 hour, DZ-24h group with diazoxide 100 μmol/L for 24 hours. Then, Western blotting was performed to detect the UQCRC1 expression. In addition, the effect of 5-HD on UQCRC1 expression in H9C2 cells was also explored. The H9C2 cells were grouped according to different treatments: control group with solvent, DZ-24h group with diazoxide 100 μmol/L for 24 hours, DZ+5-HD group with diazoxide 100 μmol/L+5-HD 100 μmol/L for 24 hours.

### Down-regulation of UQCRC1 expression on H9C2 cells by siRNA

The silencer sequence of siUQCRC1 (Primer forward, 5’-CCGUUGCUGUAGCUAACAA-3’ and Primer reverse, 5’-GGCAACGACAUCGAUUGUU-3’) was designed. Then UQCRC1 expression levels were detected 24 hours after different concentration of siUQCRC1 (0 nmol/L; 50 nmol/L; 100 nmol/L; 200 nmol/L) was transfected into H9C2 cells. And the concentration that could significantly and effectively down-regulated UQCRC1 expression levels after transfection in H9C2 cells was applied in the subsequent experiment.

### Assessment of lactate dehydrogenase (LDH) leakage and apoptosis rate after oxygen glucose deprivation (OGD) injury

The H9C2 cells were divided into three groups according to the different treatments. In the siUQCRC1 group, the silencer sequence of siUQCRC1 was transfected into H9C2 cells, while a negative control sequence was transfected into the siNC group. In the blank control group, no treatment was applied to H9C2 cells. Then, 24 hours after the transfection the OGD injury model was performed using the method of hypoxic culture as described in previous study [24], and the LDH leakage rate in the cultured medium was measured by spectrophotometry.

Furthermore, the effect of UQCRC1 down-regulation on apoptosis of H9C2 cells after OGD was explored using different treatments: Si-NC (Si-NC sequence was transfected into H9C2 cells), Si-NC+DZ-24h (Si-NC sequence was transfected into H9C2 cells and pretreated with diazoxide 100 μmol/L for 24 hours), Si-UQCRC1+DZ-24h (Si-UQCRC1 sequence was transfected). The percentage of apoptotic cells was assessed using an Apoptosis and Necrosis Assay Kit (Beyotime, China) according to the manufacturer’s instructions and a previous study [25]. The number of apoptotic or necrotic cells was analyzed using the Image pro-Plus software 5.1 and reported as the percentage of total cells.

### UQCRC1 over-expression and detection of mitochondrial membrane potential (ΔΨm)

As described in our previous study [26], the adenovirus carrying the UQCRC1 gene linked to a GFP (Ad-UQCRC1) used to infect H9C2 cells for 2 hours in 95% air and 5% CO2 at 37°C, followed by the addition of fresh medium containing FBS and the incubation was for an additional 22 hours. Then the medium was replaced 24 hours after the infection with DMEM containing 10% FBS and incubated for another 24 hours prior to treatment. The ΔΨm was assessed using confocal microscopy. Briefly, after different treatments with Ad-UQCRC1 or Ad-GFP (used as a control), the H9C2 cells were incubated with 100 nM tetramethylrhodamine ethyl ester (TMRE) (Molecular Probe) in standard Tyrode solution for 15 min and then mounted on the stage of a confocal microscope (Leica, Germany). The temperature was maintained at 37°C with a Delta T Open Dish System (Bioptechs, Butler, PA). The fluorescence intensity was determined based on computer-recorded image.

### Statistical analysis

All variables were summarized using standard descriptive statistics, such as the mean ± standard deviation (SD). An ANOVA analysis with LSD post hoc test was used to compare the differences among three or more groups, and the difference between the two groups was compared using an independent sample t test. SPSS for Windows version 17.0 (SPSS Inc., Chicago, IL) was used, and a two-tailed *P* < 0.05 was considered statistically significant.

## Results

### The effect of long-term diazoxide treatment on the delayed cardioprotection of diazoxide pretreatment in rats

After ISO injury, in rats of the group 2, serum cTnI level was significantly increased (*P* < 0.01) compared with the control group 1. (Fig 1B) After three days diazoxide pretreatment in the group 3, the serum cTnI level was significantly decreased compared to the group 2 (*P* < 0.01). Additionally, the serum cTnI level was decreased in the group 4, 5 and 6 compared with the group 2 (*P* < 0.01). However, the results showed that after 6w and 8w of diazoxide treatment, the serum cTnI level in group 7 and 8 were significantly increased compared with groups 3 to 6 (*P* < 0.01), and no difference was found between them and the group 2 (*P* > 0.05). This indicated that the effect of reducing serum cTnI levels by diazoxide pretreatment disappeared when rats were injected intraperitoneally with diazoxide for six weeks or longer. Additionally, the rat serum cTnI level in group 9 was significantly decreased compared to the groups 2, 7 and 8 (*P* < 0.01).

**Fig 2:**
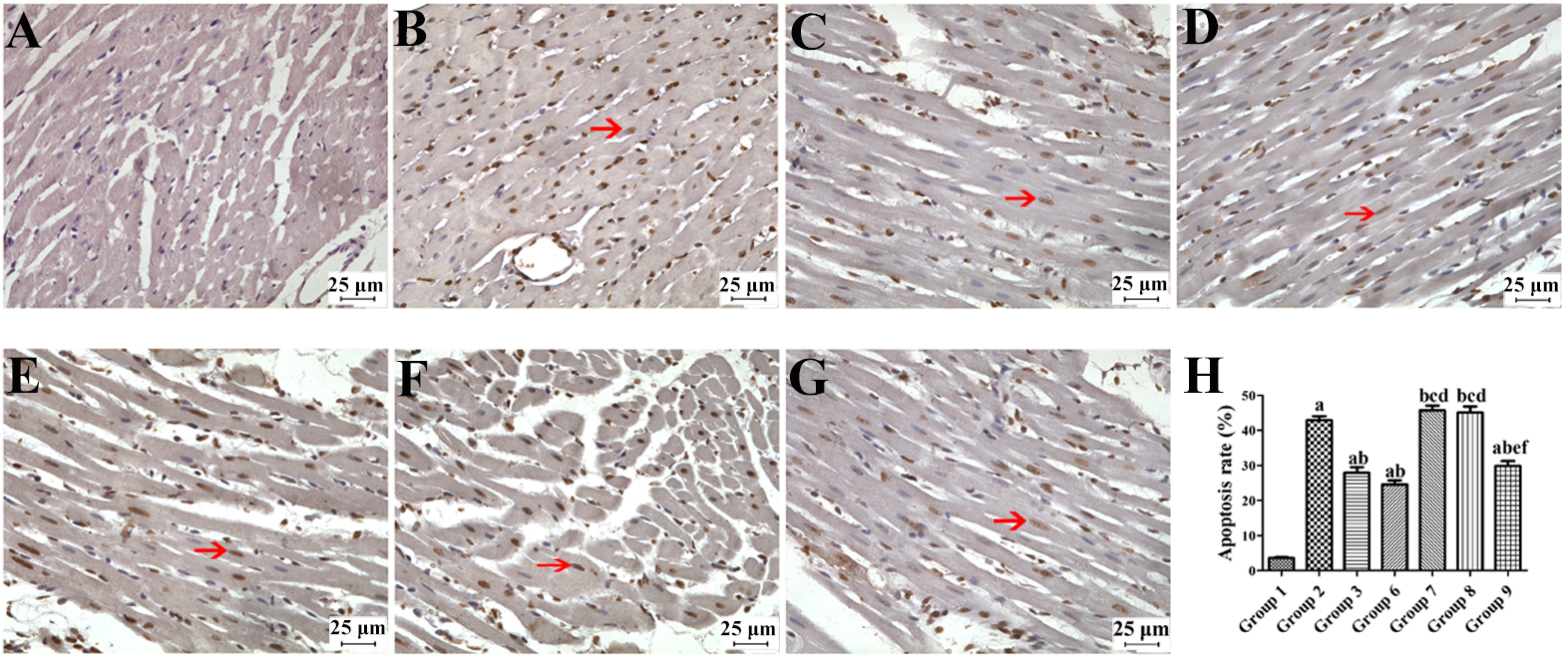
TUNEL apoptotic staining of the myocardial tissue (×200). (A) Group1; (B) Group 2; (C) Group 3; (D) Group 6; (E) Group 7; (F) Group 8; (G) Group 9; (H) Average of apoptosis rates in different groups. (Red arrow: apoptotic cell; Data were expressed as mean ± SD, n = 10; a: *P* < 0.05, *vs*. group1; b: *P* < 0.05, *vs*. group2; c: *P* < 0.05, *vs*. group3; d: *P* < 0.05, *vs*. group6; e: *P* < 0.05, *vs*. group 7; f: *P* < 0.05, *vs*. group 8).

As shown in Fig 2, apoptosis cell in the segregate myocardial tissues was determined by microscopic observation, and the average values of apoptosis rates in these groups were listed. The apoptosis rate in the group 2 was significantly increased compared with the group 1, and it was significantly decreased compared with the group 3 (*P* < 0.05). The apoptosis rates in the group 7 and 8 were increased compared with the group 3 and 6 (*P* < 0.05), but there was no difference compared with the group 2 (*P* > 0.05). In addition, the apoptosis rate in the group 9 was decreased compared with the group 7 and 8 (*P* < 0.05), and it was not different from the group 3 (*P* > 0.05).

### Proteomic analysis of myocardial MIM after long-term diazoxide treatment

**Fig 3:**
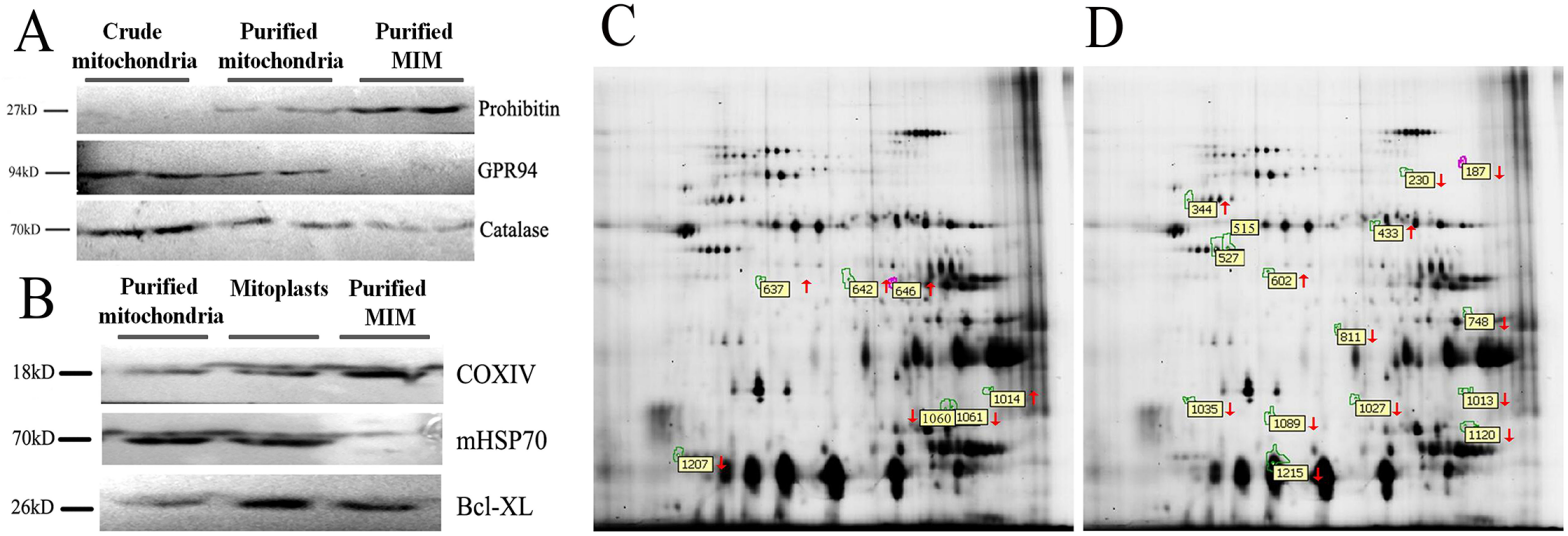
Proteomic analysis of myocardial mitochondrial inner membrane (MIM). (A) and (B) MIM purification from rat myocardial mitochondria; (C) Differential protein spots between DZ 0w group and DZ 4w group in the master gel; (D) Differential protein spots between DZ 0w group and DZ 6w group in the master gel.

As shown in Fig 3A, compared to crude mitochondria after differential centrifugation (lane 1) and purified mitochondria after consecutive Nycodenz gradients (lane 2), GPR94 and Catalase were barely detectable in the purified MIM. Additionally, mHSP70 were undetectable in the purified MIM compared to mitochondria after consecutive Nycodenz gradients and mitoplasts. However, the prohibitin and COXIV were found to be highly expressed in the purified MIM. (Fig 3B) This indicated that the purified MIM is high-purity and suitable for proteomic analysis.

Based on the analysis of Image Master software, an average of 1262 spots were detected and the average match rate of gels was 75%. In the comparison between the DZ 0w group and the DZ 4w group, seven differentially expressed protein spots were isolated by 2D-DIGE and fifteen were isolated in the comparison between DZ 0w group and the DZ 6w group (Fig 3C and 3D). Compared to the DZ 0w group 57% (4/7) of the differentially expressed protein spots were up-regulated in the DZ 4w group, while in the DZ 6w group 80% (12/15) were down-regulated.

**Fig 4:**
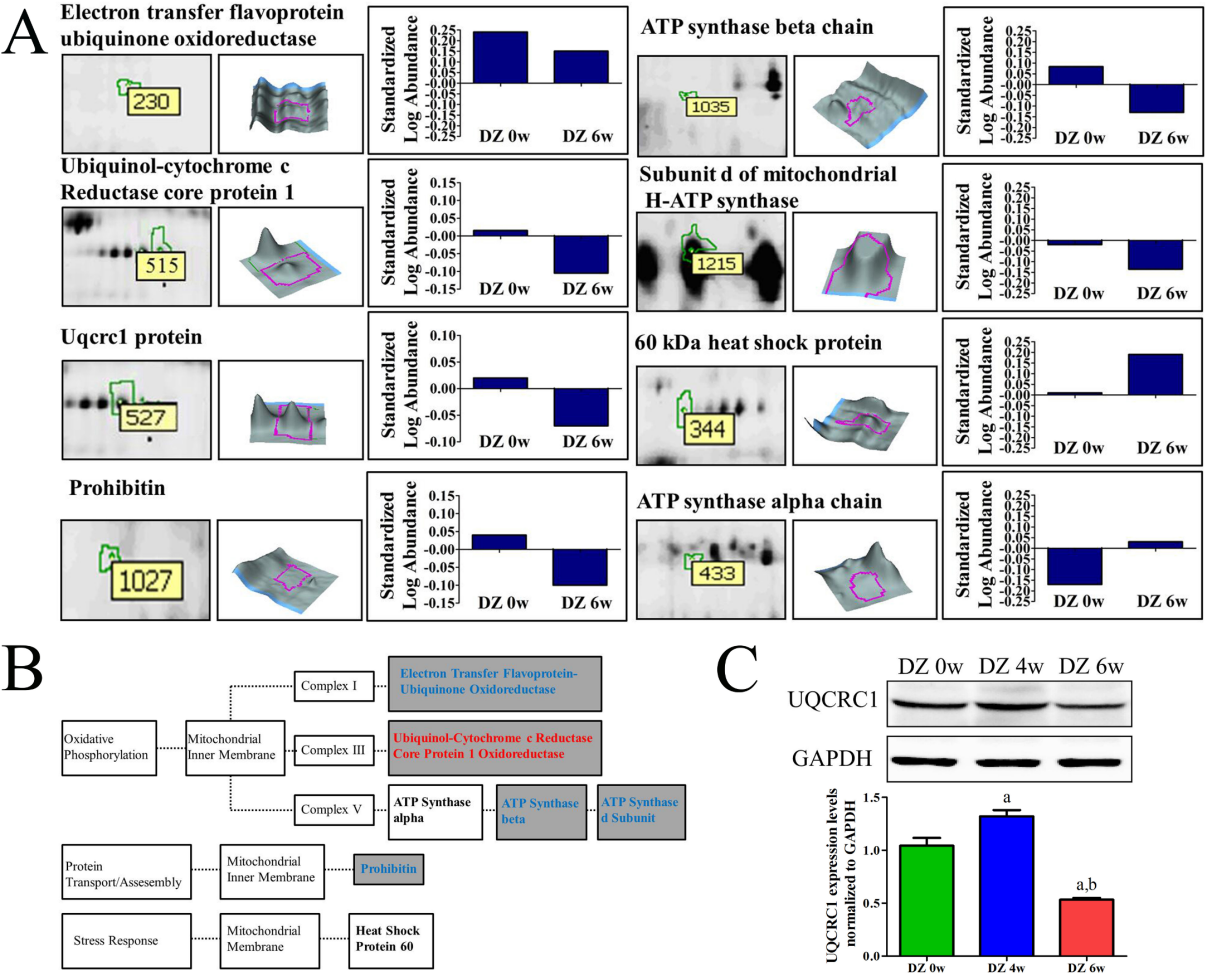
Identified proteins in DZ 6w group *vs*. DZ 0w group comparison and their functional/cellular location classification. (A) Identified differential proteins in the Group DZ 6w *vs*. DZ 0w comparison. For each identified protein, the magnified image of protein spot from the 2-DE gel is shown in left part of the panel; the 3D representation of the spot is shown in the middle part; and the graph of abundance ratio is shown in the right part. (B) Functional/cellular location classification tree of the identified proteins. Proteins were categorized by function and intracellular location. Dark gray boxes represent proteins that were down-regulated by diazoxide, and blank boxes represent proteins that were up-regulated by diazoxide. (C) Change of UQCRC1 expression in rat myocardium MIM with different time diazoxide treatment. **(**Data were expressed as mean ± SD, a: *P* < 0.05 *vs*. group DZ 0w; b: *P* < 0.05 *vs*. group DZ 4w; n = 5).

A total of eight proteins were identified in the gels by comparing the DZ 0w group with the DZ 6w group by MALDI-TOF/MS. As shown in Table 1 and Fig 4A, the identified down-regulated proteins were electron transfer flavoprotein-ubiquinone oxidoreductase (master spot no 230), ubiquinol-cytochrome c reductase core protein 1 (UQCRC1, master spot no 515), Uqcrc1 protein (master spot no527, which represented postranslational modifications of UQCRC1), prohibitin (master spot no 1027), ATP synthase beta chain (master spot no 1035), subunit d of mitochondrial H-ATP synthase (master spot no1215), while the up-regulated proteins were heat shock protein 60 (master spot no 344) and ATP synthase alpha chain (master spot no 433).

**Table 1:** Identified differential proteins in DZ 6w group *vs*. DZ 0w group comparison.

Master spot no.	T-test value	Average ratio	Accession number	Protein name	Sequence coverage (%)	Mr	P*I*
230	0.03965	-1.2207	gi|52138635	Electron transfer flavor protein-ubiquinone oxidoreductase	29.71%	68198.11	7.33
515	0.03512	-1.2804	gi|51259340	Ubiquinol-cytochrome c reductase core protein 1	7%	53500	5.57
527	0.01304	-1.2254	gi|20988752	Uqcrc1 protein	8%	28872	6.08
1027	0.02874	-1.8868	gi|13937353	Prohibitin	7.91%	27716.7	5.44
1035	0.02112	-1.3885	gi|54792127	ATP synthase beta chain	9.45%	56353.6	5.18
1215	0.005843	-1.3082	gi|220904	Subunit d of mitochondrial H-ATP synthase	18%	18827	5.78
344	0.003429	1.4915	gi|206597443	60kDa heat shock protein	16.4%	60955.29	5.91
433	0.04913	1.5882	gi|40538742	ATP synthase alpha chain	27.31%	59753.66	9.22

Furthermore, the change of UQCRC1, a subunit of complex III (Fig 4B), expression in different treatment groups was validated by western blotting analysis. As shown in Fig 4C. The results of Western blotting analysis showed that UQCRC1 expression in the rat mitochondria sample of the group DZ 4w was increased compared with group DZ 0w (*P* < 0.05). UQCRC1 expression in the rat mitochondria sample of the group DZ 6w was decreased compared with both the group DZ 0w (*P* < 0.05) and group DZ 4w (*P* < 0.05).

### Change in UQCRC1 expression in H9C2 cells

**Fig 5:**
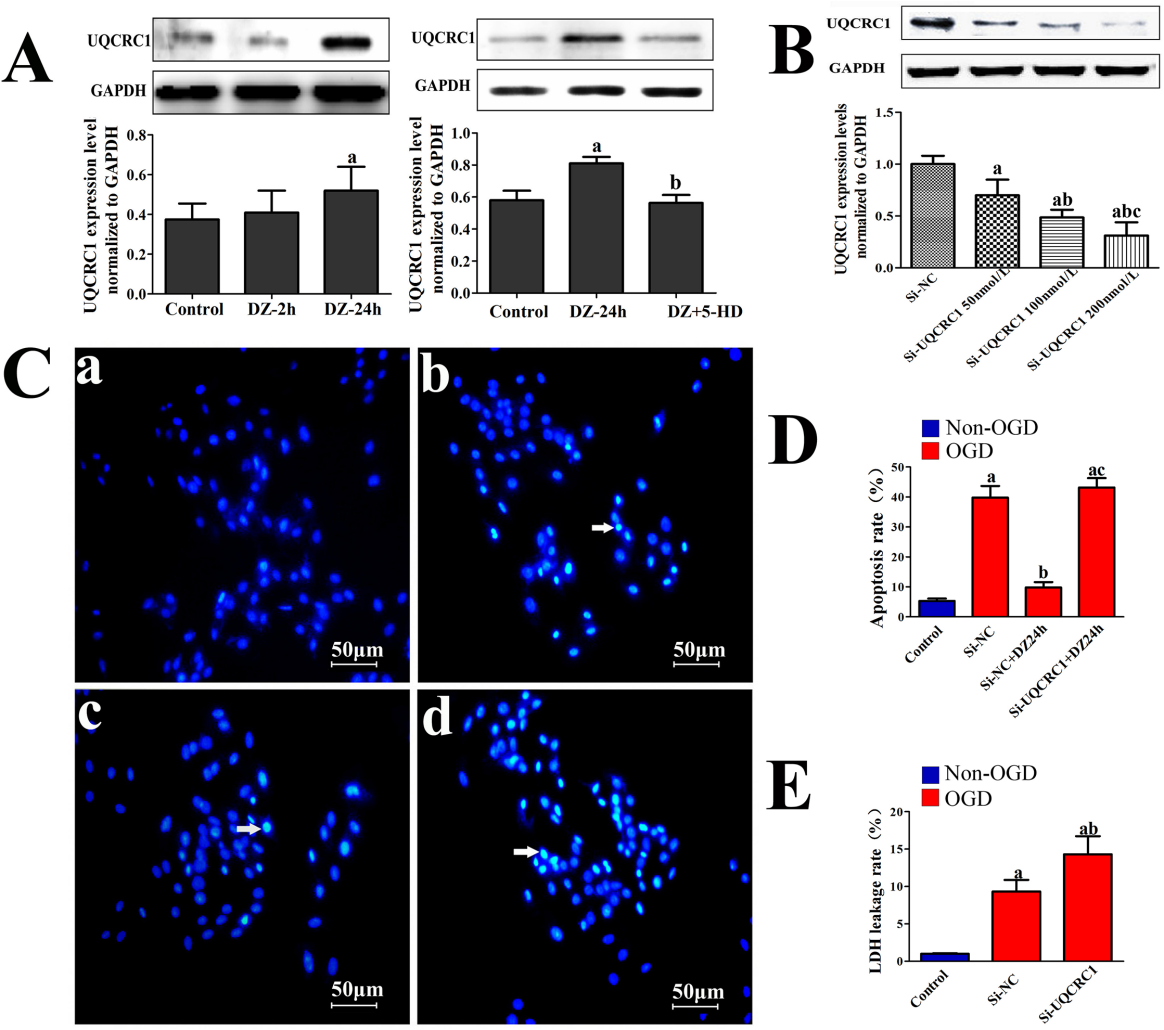
The effect of diazoxide treatment on UQCRC1 expression and the effects of UQCRC1down-regulation on apoptosis rate and LDH leakage rate after OGD in H9C2 cells. (A) Change in UQCRC1 expression after treatment with diazoxide or diazoxide combined with 5-HD (n = 8); (B) The effect of silencer sequence of siUQCRC1 on UQCRC1 expression levels in H9C2 cells (n = 6); (C) and (D) The effect of UQCRC1down-regulation on apoptosis rate of H9C2 cells after OGD (n = 5); (E) The effect of UQCRC1down-regulation on LDH leakage of H9C2 cells after OGD (n = 8). White arrow: apoptotic cell; Data were expressed as mean ± SD; a, *P* < 0.05, *vs*. first group; b, *P* < 0.05, *vs*. second group; c, *P* < 0.05, *vs*. third group.

As shown in Fig 5A, UQCRC1 expression in H9C2 cells after 24 h diazoxide pretreatment in SWOP group (approximately 1.4-folds, *P* < 0.05) was significantly increased compared with the control group. This indicated that diazoxide pretreatment could up-regulate UQCRC1 expression. However, the results showed that UQCRC1 expression in H9C2 cells of the (DZ+5HD)-SWOP group was significantly decreased compared with the DZ-SWOP group (*P* < 0.05, Fig 5A), indicating that the up-regulated level of UQCRC1 expression was abrogated when 5-HD was given along with diazoxide pretreatment.

### The effect of down-regulation of UQCRC1 expression on H9C2 cells after OGD injury

The UQCRC1 expression levels were significantly down-regulated when applied Si-UQCRC1 at the concentration of 50 nmol/L, 100 nmol/L and 200 nmol/L by about 30%, 50% and 70% compared to Si-NC respectively. (Fig 5B). Therefore, 200nmol/L was identified as significantly down-regulating UQCRC1 expression after transfection in H9C2 cells and was applied in the subsequent experiment. Apoptotic H9C2 cells were observed after OGD injury (Fig 5C), and the apoptosis rate decreased in Si-NC+DZ-24h group compared to Si-NC group. (Fig 5D, *P* < 0.05) But the apoptosis rate in Si-QUCRC1+DZ-24h group significantly increased compared to Si-NC+DZ-24h group (Fig 5D, *P* < 0.05), and it was not different from the Si-NC group. (Fig 5D, *P* > 0.05) And compared to the siNC group, the LDH leakage rate was significantly higher in the siUQCRC1 group. (*P* < 0.05, Fig 5E)

### The effect of UQCRC1 over-expression on ΔΨm

**Fig 6:**
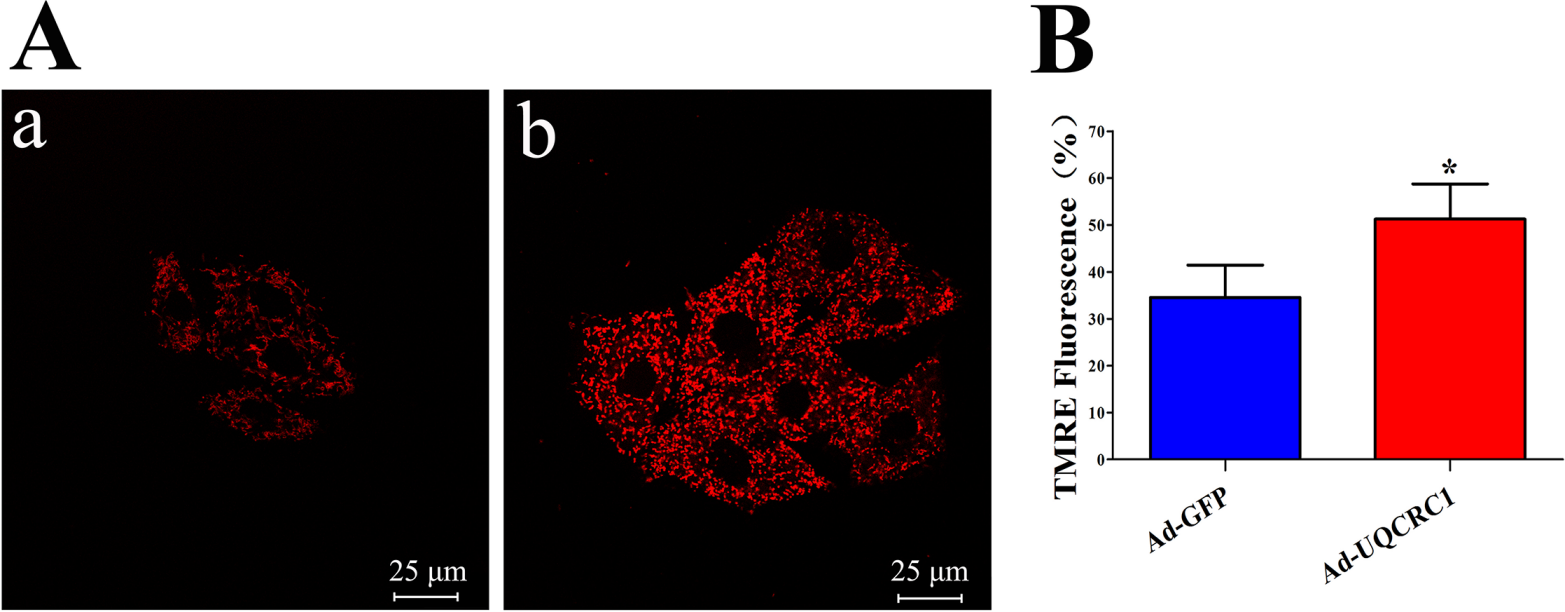
The effect of UQCRC1 over-expression on mitochondrial membrane potential. (A) Confocal fluorescence images of TMRE (a: Ad-GFP, b: Ad-UQCRC1); (B) Mitochondrial membrane potential of H9C2 cells assessed by TMRE (Summarized data for TMRE fluorescence intensity, * *P* < 0.05 *vs*. Ad-GFP, n = 5; Data are expressed as the means ± SD).

The ΔΨm of H9C2 cells was assessed using tetra methyl rhodamine ethyl ester (TMRE), and the confocal fluorescence images of TMRE were showed in Fig 6A. And as shown in Fig 6B, UQCRC1 over-expression significantly increase the ΔΨm of H9C2 cells in Ad-QUCRC1 group compared to that in Ad-GFP group.

## Discussion

The current study used a rat model of long-term treatment with diazoxide to establish a loss-of-cardioprotection model. The results showed that after diazoxide treatment for 6w or longer, the rat serum cTnI level was significantly higher than in non-diazoxide treated rats when exposed to ISO myocardial injury even if diazoxide pretreatment was applied. This indicated that the effect of delayed myocardial protection of preconditioning by diazoxide pretreatment disappeared when rats were intraperitoneally injected with diazoxide for six weeks or longer. However, the loss-of-cardioprotection could be avoided by being given 5-HD, a mitoK_ATP_ channel blocker [18, 19], together with diazoxide. This phenomenon further validated that the current model could successfully mimic loss-of-cardioprotection by long-term mitoK_ATP_ opening. Therefore, the current model has the potential to serve as a strategy to investigate the functional proteins that are differently expressed at baseline after long-term diazoxide treatment and are involved in delayed cardioprotection.

For exploring the possible differently expressed functional proteins at baseline, proteomic analysis was then performed in the current study. The results showed that 57% (4/7) of differentially expressed protein spots were up-regulated after four weeks diazoxide treatment, when the cardioprotection effect of diazoxide preatment was still present. However, 15 differentially expressed protein spots, of which 80% (12/15) were down-regulated after six weeks of diazoxide treatment, when loss-of-cardioprotection was found to be present. This indicated that while the cardioprotection effect of preconditioning was induced by up-regulation of functional proteins, the loss-of-cardioprotection effect of long-term diazoxide treatment might be caused by down-regulation of some functional proteins. In particular, among these identified proteins ATP synthase alpha and beta subunits are changed in opposite directions. This indicated that even if both the ATP synthase alpha and beta subunits are components of complex V, the response to pharmacological treatment would not be consistent. This phenomenon has been also demonstrated by a previous study [27], Through proteomics analysis, the study found that expression of ATP synthase alpha chain was found to be altered by ischemic preconditioning but ATP synthase beta chain not, while expression of ATP synthase beta chain was altered by adenosine but not for ATP synthase alpha chain.

Through proteomics analysis, several proteins which exclusively located on MIM were identified and most of them were components of complex proteins that are associated with oxidative phosphorylation in the mitochondrial respiratory chain (Fig 4B) [28]. Among these down-regulated proteins, UQCRC1 is a subunit of complex III, which was one of the main sources of reactive oxygen species (ROS) [29]. And ROS has been demonstrated to play an important role in ischemic preconditioning heart tissue [30, 31]. Based on previous reports and the results of the current study, we speculated that UQCRC1 might be the most potentially effective protein involved in delayed myocardial protection of preconditioning induced by diazoxide pretreatment, and thus we preferentially explore the possible role of UQCRC1. Furthermore, in an invitro model of H9C2 cells, we found that UQCRC1 was up-regulated after diazoxide late preconditioning, and the up-regulation could be abrogated by the mitoK_ATP_ channel blocker 5-HD. On the basis of these studies, it was speculated that UQCRC1 might be involved in events downstream of the mitoK_ATP_ action loop and related to the effect of delayed cardioprotection induced by diazoxide.

UQCRC1 was found to be increased in tumor tissues and might contribute to the development of hepatocellular carcinomas tumors [32]. A previous study has demonstrated that UQQCRC1 gene over-expression can enhance complex III activity in neuroblastoma cells, and therefore may contribute to a severe neurological disorder [33]. Furthermore, decreased UQCRC1 expression levels in a mitochondrial sample isolated from hearts was found to possibly contribute to contractile dysfunction in diabetic mice [34]. Recently, mitochondrial dysfunction was also associated with a specific loss of UQCRC1 in cells of epithelial origin [35] and mouse spermatocytes [36]. The current results further showed that UQCRC1 over-expression significantly increase the ΔΨm, an indicator of mitochondrial function. Based on these findings, we speculated that UQCRC1 can regulate of mitochondrial function and its specific role in myocardial cell needs to be explored in the further study.

Although the previous studies support that UQCRC1 expression contributes to mitochondrial function, at present no study has directly explored the function of UQCRC1 in cardioprotection. Thus, in the current study, we also performed an exploratory study to investigate the effects of down-regulation of UQCRC1 on H9C2 cells after OGD injury. Interestingly, the results showed that the LDH leakage rate was significantly increased in the H9C2 cells with UQCRC1 down-expression after OGD injury. More important, we found that diazoxide treatment could significantly decrease the apoptosis rate, while such anti-apoptosis effect could be abrogated by UQCRC1 down-regulation. Therefore, these results strengthen the hypothesis that UQCRC1 can serve as a targeted molecule site in cardioprotection, and anti-apoptosis might be its underlying mechanisms.

Several limitations should be considered when interpreting the results of the current study. First, in the current study, an ISO model was used to mimic myocardial ischemia in rat myocardium. Different from the infarction model induced by ligation of anterior descending arteries or the ischemia reperfusion model in vitro, ISO injection produces an 'infarct-like' myocardial injury by positive inotropic effect [37]. Second, based on the previous studies UQCRC1 might be the most potentially effective protein involved in delayed cardioprotection, and thus we preferentially chose UQCRC1 to investigate and validate its effect in cardioprotection. Although the current results regarding the other proteins could provide some evidence for their possible effect in cardioprotection, it needed further validation in the future. Thirdly, in the study the role of UQCRC1 expression in cardioprotection was only measured in H9C2 cells after OGD injury. Therefore, a more comprehensive and specific assessment of UQCRC1, e.g., whether the changes of UQCRC1 abundance affect the enzymatic activities of Complex III, or mitoK_ATP_ channel, is required in the future.

## Conclusions

In conclusion, the current study found that long-term administration of diazoxide can abolish the ability of the delayed cardioprotection effect induced by diazoxide preconditioning. The proteomic approach identified several differentially expressed proteins, including down-regulated UQCRC1, which is located in the MIM and is linked closely to the mitochondrial K_ATP_ channel. Also, UQCRC1 was found to increase after diazoxide pretreatment, while this effect was abrogated by 5-HD administration. Additionally, UQCRC1 expression was found to play an important role in regulating the LDH leakage and apoptosis rate in H9C2 cells after OGD injury. Therefore, UQCRC1 might be an important molecular site for pharmacological preconditioning.

## Supporting information

S1 Checklist ARRIVE guidelines checklist. (PDF)

S1 Data Minimal data set. (ZIP)
