# Supplementary material for: Ubiquinol-cytochrome c reductase core protein 1 may be involved in delayed cardioprotection from preconditioning induced by diazoxide
Source: PLoS One. 2017 Jul 27;12(7):e0181903. doi: 10.1371/journal.pone.0181903 (PMC5531499; doi:10.1371/journal.pone.0181903)
Supplement: S1 Data — (ZIP) [file pone.0181903.s002.zip › S1 ZIP. Minimal data set/proteomics Data/Group 0w and 4w/12.htm]

xml version="1.0"?

## Proteins of interest

##### File: D:\DIGE project\060605-mito\mito\BVA\12\12.xml

**Experiment data**

**Image data**

**Gel data**

**Protein data**

---

### Experiment data

|  |  |
| --- | --- |
| Image | No. of included spots |
| gel1-standard-cy2.gel | 1121 |
| gel1-cy3.gel | 1121 |
| gel1-cy5.gel | 1121 |
| gel2-standard-cy2.gel | 1336 |
| gel2-cy3.gel | 1336 |
| gel2-cy5.gel | 1336 |
| gel3-standard cy2.gel | 1328 |
| gel3-cy3.gel | 1328 |
| gel3-cy5.gel | 1328 |

**Back to top**

---

### Image data

|  |  |  |  |  |  |  |  |  |  |  |  |  |  |  |  |  |  |  |
| --- | --- | --- | --- | --- | --- | --- | --- | --- | --- | --- | --- | --- | --- | --- | --- | --- | --- | --- |
| Master spot no. | Volume | | | | | | | | | Peak Height | | | | | | | | |
|  | gel1-standard-cy2.gel | gel1-cy3.gel | gel1-cy5.gel | gel2-standard-cy2.gel | gel2-cy3.gel | gel2-cy5.gel | gel3-standard cy2.gel | gel3-cy3.gel | gel3-cy5.gel | gel1-standard-cy2.gel | gel1-cy3.gel | gel1-cy5.gel | gel2-standard-cy2.gel | gel2-cy3.gel | gel2-cy5.gel | gel3-standard cy2.gel | gel3-cy3.gel | gel3-cy5.gel |
| 637 | 265566 | 175078 | 302447 | 227915 | 329666 | 192964 | 32559 | 91653 | 46975 | 939 | 576 | 1021 | 764 | 1088 | 618 | 99 | 334 | 159 |
| 642 | 380713 | 467505 | 601718 | 804289 | 1118675 | 911780 | 93423 | 320125 | 217846 | 782 | 998 | 1234 | 1529 | 1906 | 1601 | 217 | 749 | 574 |
| 646 | 1218637 | 775508 | 2845821 | 205312 | 286585 | 255001 | 109593 | 446851 | 167797 | 4065 | 2469 | 9315 | 769 | 1047 | 949 | 429 | 1619 | 785 |
| 1014 | 705694 | 1115998 | 1124346 | 257634 | 490345 | 403549 | 47915 | 209293 | 142766 | 1853 | 2735 | 2862 | 866 | 1549 | 1322 | 123 | 613 | 476 |
| 1060 | 764480 | 834026 | 1108985 | 755760 | 663878 | 463813 | 2994075 | 4927555 | 6267312 | 1793 | 1468 | 2648 | 1713 | 1506 | 884 | 7290 | 13147 | 17254 |
| 1061 | 533584 | 496004 | 648484 | 388237 | 348730 | 242226 | 1238135 | 2091257 | 2425385 | 1215 | 1217 | 1732 | 1220 | 1082 | 710 | 3878 | 6506 | 7995 |
| 1207 | 608741 | 1013814 | 912842 | 447784 | 967167 | 383118 | 209490 | 587721 | 741819 | 1462 | 2601 | 2433 | 1271 | 3161 | 785 | 338 | 1026 | 1558 |

**Back to top**

---

### Gel data

|  |  |  |  |
| --- | --- | --- | --- |
| Master spot no. | Match confidence | | |
|  | D:\DIGE project\060605-mito\mito\DIA\gel1.dia | D:\DIGE project\060605-mito\mito\DIA\gel2.dia | D:\DIGE project\060605-mito\mito\DIA\gel3.dia |
|  |  |  |  |
| 637 | AUTO\_2 | AUTO\_1 | AUTO\_2 |
| 642 | AUTO\_2 | AUTO\_1 | AUTO\_2 |
| 646 | AUTO\_2 | AUTO\_1 | AUTO\_2 |
| 1014 | AUTO\_2 | AUTO\_1 | AUTO\_2 |
| 1060 | AUTO\_2 | AUTO\_1 | AUTO\_2 |
| 1061 | AUTO\_2 | AUTO\_1 | AUTO\_2 |
| 1207 | AUTO\_2 | AUTO\_1 | AUTO\_2 |

**Back to top**

---

### Protein data

|  |  |  |
| --- | --- | --- |
| Master spot no. | T-test value | Average ratio |
| 637 | 0.04035 | 1.5666 |
| 642 | 0.01914 | 1.1528 |
| 646 | 0.02365 | 2.2460 |
| 1014 | 0.01743 | 1.1927 |
| 1060 | 0.009408 | -1.5182 |
| 1061 | 0.03989 | -1.3289 |
| 1207 | 0.02767 | -1.5832 |

**Back to top**
