# Supplementary material for: Ubiquinol-cytochrome c reductase core protein 1 may be involved in delayed cardioprotection from preconditioning induced by diazoxide
Source: PLoS One. 2017 Jul 27;12(7):e0181903. doi: 10.1371/journal.pone.0181903 (PMC5531499; doi:10.1371/journal.pone.0181903)
Supplement: S1 Data — (ZIP) [file pone.0181903.s002.zip › S1 ZIP. Minimal data set/proteomics Data/Group 0w and 6w/13.htm]

xml version="1.0"?

## Proteins of interest

##### File: D:\DIGE project\060605-mito\mito\BVA\13\13.xml

**Experiment data**

**Image data**

**Gel data**

**Protein data**

---

### Experiment data

|  |  |  |
| --- | --- | --- |
| Image | No. of included spots | No. of matched spots |
| gel1-standard-cy2.gel | 1121 | 866 |
| gel1-cy3.gel | 1121 | 866 |
| gel1-cy5.gel | 1121 | 866 |
| gel2-standard-cy2.gel | 1336 | 1336 |
| gel2-cy3.gel | 1336 | 1336 |
| gel2-cy5.gel | 1336 | 1336 |
| gel3-standard cy2.gel | 1328 | 640 |
| gel3-cy3.gel | 1328 | 640 |
| gel3-cy5.gel | 1328 | 640 |

**Back to top**

---

### Image data

|  |  |  |  |  |  |  |  |  |  |  |  |  |  |  |  |  |  |  |
| --- | --- | --- | --- | --- | --- | --- | --- | --- | --- | --- | --- | --- | --- | --- | --- | --- | --- | --- |
| Master spot no. | Volume | | | | | | | | | Peak Height | | | | | | | | |
|  | gel1-standard-cy2.gel | gel1-cy3.gel | gel1-cy5.gel | gel2-standard-cy2.gel | gel2-cy3.gel | gel2-cy5.gel | gel3-standard cy2.gel | gel3-cy3.gel | gel3-cy5.gel | gel1-standard-cy2.gel | gel1-cy3.gel | gel1-cy5.gel | gel2-standard-cy2.gel | gel2-cy3.gel | gel2-cy5.gel | gel3-standard cy2.gel | gel3-cy3.gel | gel3-cy5.gel |
| 187 | 258686 | 585998 | 424758 | 110439 | 152460 | 120847 | 38305 | 163123 | 154868 | 606 | 1534 | 979 | 525 | 829 | 620 | 45 | 304 | 361 |
| 230 | 257697 | 500984 | 469520 | 225423 | 393037 | 295091 | 38345 | 174279 | 148204 | 948 | 1704 | 1868 | 699 | 1192 | 851 | 127 | 657 | 563 |
| 344 | 768501 | 912259 | 1466344 | 849027 | 1626981 | 941911 | 45693 | 111890 | 100301 | 3384 | 3857 | 5674 | 2291 | 4645 | 3119 | 160 | 351 | 369 |
| 433 | 786619 | 539482 | 1047834 | 731967 | 991420 | 615363 | 43330 | 148697 | 70836 | 1919 | 1239 | 2857 | 1948 | 2901 | 1710 | 125 | 536 | 208 |
| 515 | 1920092 | 2169100 | 1798470 | 2532306 | 2662466 | 1998860 | 280331 | 533280 | 636068 | 7537 | 6846 | 5698 | 9055 | 7100 | 7071 | 1435 | 2390 | 2903 |
| 527 | 5320901 | 6213359 | 5682437 | 5288358 | 5642244 | 5014183 | 520147 | 1067919 | 1208441 | 21928 | 21238 | 21344 | 22510 | 17490 | 20066 | 1964 | 4180 | 4083 |
| 602 | 385959 | 217346 | 419373 | 296057 | 335395 | 124960 | 28029 | 101323 | 22841 | 1258 | 664 | 1541 | 1222 | 1308 | 518 | 80 | 432 | 69 |
| 748 | 331544 | 500638 | 370123 | 387620 | 450348 | 295384 | 323030 | 1319082 | 1095297 | 975 | 1527 | 1090 | 1162 | 1453 | 957 | 843 | 3821 | 3092 |
| 811 | 614693 | 714991 | 414561 | 161004 | 118438 | 107681 | 106016 | 238116 | 275854 | 1058 | 1214 | 711 | 621 | 413 | 423 | 269 | 561 | 705 |
| 1013 | 182866 | 310971 | 203149 | 307524 | 342238 | 311990 | 49849 | 181953 | 202251 | 589 | 1237 | 831 | 652 | 818 | 789 | 157 | 952 | 921 |
| 1027 | 375207 | 443217 | 365412 | 348094 | 345994 | 332105 | 31124 | 98919 | 77796 | 1008 | 1243 | 973 | 939 | 1001 | 1056 | 161 | 532 | 415 |
| 1035 | 220254 | 319066 | 187001 | 190736 | 182427 | 92556 | 31144 | 62922 | 80045 | 1006 | 1258 | 1024 | 1095 | 711 | 293 | 104 | 291 | 326 |
| 1089 | 92686 | 108242 | 65399 | 191985 | 153784 | 215246 | 53260 | 97422 | 112805 | 359 | 407 | 222 | 517 | 346 | 572 | 109 | 295 | 304 |
| 1120 | 965527 | 1144868 | 927421 | 1018769 | 928875 | 797321 | 197851 | 831154 | 472245 | 1576 | 1907 | 993 | 2830 | 2643 | 2549 | 326 | 1823 | 920 |
| 1215 | 51009408 | 55811659 | 44934817 | 30927250 | 28827117 | 31880889 | 735726 | 762381 | 1498138 | 56242 | 57915 | 61335 | 55710 | 57826 | 60865 | 1849 | 1834 | 3924 |

**Back to top**

---

### Gel data

|  |  |  |  |
| --- | --- | --- | --- |
| Master spot no. | Match confidence | | |
|  | D:\DIGE project\060605-mito\mito\DIA\gel1.dia | D:\DIGE project\060605-mito\mito\DIA\gel2.dia | D:\DIGE project\060605-mito\mito\DIA\gel3.dia |
|  |  |  |  |
| 187 | AUTO\_2 | AUTO\_1 | AUTO\_2 |
| 230 | AUTO\_2 | AUTO\_1 | AUTO\_2 |
| 344 | AUTO\_2 | AUTO\_1 | AUTO\_2 |
| 433 | AUTO\_2 | AUTO\_1 | AUTO\_2 |
| 515 | AUTO\_2 | AUTO\_1 | AUTO\_1 |
| 527 | AUTO\_1 | AUTO\_1 | AUTO\_1 |
| 602 | AUTO\_2 | AUTO\_1 | AUTO\_2 |
| 748 | AUTO\_2 | AUTO\_1 | AUTO\_2 |
| 811 | AUTO\_2 | AUTO\_1 | AUTO\_2 |
| 1013 | AUTO\_2 | AUTO\_1 | AUTO\_2 |
| 1027 | AUTO\_2 | AUTO\_1 | AUTO\_2 |
| 1035 | AUTO\_2 | AUTO\_1 | AUTO\_2 |
| 1089 | AUTO\_2 | AUTO\_1 | AUTO\_2 |
| 1120 | AUTO\_2 | AUTO\_1 | AUTO\_2 |
| 1215 | AUTO\_2 | AUTO\_1 | AUTO\_2 |

**Back to top**

---

### Protein data

|  |  |  |
| --- | --- | --- |
| Master spot no. | T-test value | Average ratio |
| 187 | 0.04999 | -1.5891 |
| 230 | 0.03965 | -1.2207 |
| 344 | 0.003429 | 1.4915 |
| 433 | 0.04913 | 1.5882 |
| 515 | 0.03512 | -1.2804 |
| 527 | 0.01304 | -1.2254 |
| 602 | 0.03643 | 2.0418 |
| 748 | 0.02793 | -1.5826 |
| 811 | 0.01409 | -1.9663 |
| 1013 | 0.02874 | -1.8868 |
| 1027 | 0.02112 | -1.3885 |
| 1035 | 0.01237 | -1.7002 |
| 1089 | 0.012 | -1.6601 |
| 1120 | 0.01732 | -1.4268 |
| 1215 | 0.005843 | -1.3082 |

**Back to top**
