# Supplementary material for: Ubiquinol-cytochrome c reductase core protein 1 may be involved in delayed cardioprotection from preconditioning induced by diazoxide
Source: PLoS One. 2017 Jul 27;12(7):e0181903. doi: 10.1371/journal.pone.0181903 (PMC5531499; doi:10.1371/journal.pone.0181903)
Supplement: S1 Data — (ZIP) [file pone.0181903.s002.zip › S1 ZIP. Minimal data set/proteomics Data/Group 0w and 4w/report1-group12-table report mito.doc]

Report1-geoup12-table report mito

Protein Table	T-test and Av.Ratio: 2 / 1

Pos.	Master No.	T-test	Av. Ratio

1	1207	0.028	-1.58

2	1060	0.0094	-1.52

3	1061	0.040	-1.33

4	642	0.019	1.15

5	1014	0.017	1.19

6	637	0.040	1.57

7	646	0.024	2.25
