# Supplementary material for: Ubiquinol-cytochrome c reductase core protein 1 may be involved in delayed cardioprotection from preconditioning induced by diazoxide
Source: PLoS One. 2017 Jul 27;12(7):e0181903. doi: 10.1371/journal.pone.0181903 (PMC5531499; doi:10.1371/journal.pone.0181903)
Supplement: S1 Data — (ZIP) [file pone.0181903.s002.zip › S1 ZIP. Minimal data set/proteomics Data/Group 0w and 4w/report2-group12-graph view mito.doc]

Report2-group12-graph view report mito

Master NO:1207

Master NO:1060

Master NO:1061

Master NO:642

Master NO:1014

Master NO:637

Master NO:646
