# Supplementary material for: Ubiquinol-cytochrome c reductase core protein 1 may be involved in delayed cardioprotection from preconditioning induced by diazoxide
Source: PLoS One. 2017 Jul 27;12(7):e0181903. doi: 10.1371/journal.pone.0181903 (PMC5531499; doi:10.1371/journal.pone.0181903)
Supplement: S1 Data — (ZIP) [file pone.0181903.s002.zip › S1 ZIP. Minimal data set/proteomics Data/Group 0w and 6w/report1-group13-table report mito.doc]

Report1-geoup12-table report mito

Protein Table	T-test and Av.Ratio: 2 / 1

Pos.	Master No.	T-test	Av. Ratio

1	1207	0.028	-1.58

2	1060	0.0094	-1.52

3	1061	0.040	-1.33

4	642	0.019	1.15

5	1014	0.017	1.19

6	637	0.040	1.57

7	646	0.024	2.25

Report1-group13-table report mito

Protein Table	T-test and Av.Ratio: 3 / 1

Pos.	Master No.	T-test	Av. Ratio

1	811	0.014	-1.97

2	1013	0.029	-1.89

3	1035	0.012	-1.70

4	1089	0.012	-1.66

5	187	0.050	-1.59

6	748	0.028	-1.58

7	1120	0.017	-1.43

8	1027	0.021	-1.39

9	1215	0.0058	-1.31

10	515	0.035	-1.28

11	527	0.013	-1.23

12	230	0.040	-1.22

13	344	0.0034	1.49

14	433	0.049	1.59

15	602	0.036	2.04

Report1-group23-table report mito

Protein Table	T-test and Av.Ratio: 3 / 2

Pos.	Master No.	T-test	Av. Ratio

1	1087	0.0081	-1.69

2	914	0.022	-1.64

3	877	0.0084	-1.60

4	924	0.028	-1.45

5	815	0.018	-1.44

6	1027	0.040	-1.39

7	1023	0.042	-1.36

8	1078	0.012	-1.33

9	1214	0.019	-1.25

10	336	0.014	1.21

11	524	0.032	1.39

12	557	0.049	1.51

13	796	0.020	1.59
