# Supplementary material for: Ubiquinol-cytochrome c reductase core protein 1 may be involved in delayed cardioprotection from preconditioning induced by diazoxide
Source: PLoS One. 2017 Jul 27;12(7):e0181903. doi: 10.1371/journal.pone.0181903 (PMC5531499; doi:10.1371/journal.pone.0181903)
Supplement: S1 Data — (ZIP) [file pone.0181903.s002.zip › S1 ZIP. Minimal data set/proteomics Data/Group 0w and 6w/report2-group13-graph report mito.doc]

Report2-group13-graph report mito

Master NO:811

Master NO:1013

Master NO:1035

Master NO:1089

Master NO:187

Master NO:748

Master NO:1120

Master NO:1027

Master NO:1215

Master NO:515

Master NO:527

Master NO:230

Master NO:344

Master NO:433

Master NO:602
